# Supplementary material for: From sequence to enzyme mechanism using multi-label machine learning
Source: BMC Bioinformatics. 2014 May 19;15:150. doi: 10.1186/1471-2105-15-150 (PMC4229970; doi:10.1186/1471-2105-15-150)
Supplement: Additional file 2 — Java code of ml2db. Additional file ml2db_code.tar.gz contains the Java source code to run the multi-label machine learning experiments and save the results to database. The code’s Javadoc is included. [file 1471-2105-15-150-S2.zip › additional file 2/ml2db/ml2db/doc/main/ml2db/mulan/learn/traintest/TrainTestInputManager.html]

TrainTestInputManager


JavaScript is disabled on your browser.


- Overview
- Package
- Class
- Use
- Tree
- Deprecated
- Index
- Help

- Prev Class
- Next Class

- Frames
- No Frames

- All Classes

- Summary:
- Nested |
- Field |
- Constr |
- Method

- Detail:
- Field |
- Constr |
- Method


main.ml2db.mulan.learn.traintest

## Class TrainTestInputManager

- java.lang.Object
- - main.ml2db.mulan.learn.crosseval.CrossInputManager
  - - main.ml2db.mulan.learn.traintest.TrainTestInputManager

- ---

    

  ```
  public class TrainTestInputManager
  extends CrossInputManager
  ```

  An input manager that gets both the training and testing instances from a
  database arff record identifier.

  Version:
  :   13 Jul 2011

  Author:
  :   Luna De Ferrari ldf(at)st-andrews.ac.uk

- - ### Constructor Summary

    Constructors

    Constructor and Description

    `TrainTestInputManager(IdMultiLabelInstances trainSet,
    IdMultiLabelInstances testSet,
    TrainTestExperimenter experimenter)`

    Gets the instances as object.

    `TrainTestInputManager(IdMultiLabelInstances trainSet,
    TrainTestExperimenter experimenter)`

    Gets the instances as object.

    `TrainTestInputManager(int trainArffRecordId,
    DbManagers dbMan,
    TrainTestExperimenter trainTestExperimenter)`

    `TrainTestInputManager(int trainArffRecordId,
    int testArffRecordId,
    DbManagers dbMan,
    TrainTestExperimenter experimenter)`
  - ### Method Summary

    Methods

    | Modifier and Type | Method and Description |
    | --- | --- |
    | `void` | `checkTestArffRecord()` |
    | `MulanArffRecord` | `getTestArffRecord()` |
    | `IdMultiLabelInstances` | `getTestDataSet()` |
    | `void` | `setTestArffRecord(int testArffId, DbManagers dbMan)` |
    | `void` | `setTestDataSet(IdMultiLabelInstances testSet)` |

    - ### Methods inherited from class main.ml2db.mulan.learn.crosseval.CrossInputManager

      `checkTrainArffRecord, getDbManagers, getExperiment, getTrainArffPath, getTrainArffRecord, getTrainDataSet, getTrainXmlPath, setTrainDataSet`
    - ### Methods inherited from class java.lang.Object

      `equals, getClass, hashCode, notify, notifyAll, toString, wait, wait, wait`

- - ### Constructor Detail


    - #### TrainTestInputManager

      ```
      public TrainTestInputManager(IdMultiLabelInstances trainSet,
                           IdMultiLabelInstances testSet,
                           TrainTestExperimenter experimenter)
      ```

      Gets the instances as object.

      Parameters:
      :   `trainSet` -


    - #### TrainTestInputManager

      ```
      public TrainTestInputManager(IdMultiLabelInstances trainSet,
                           TrainTestExperimenter experimenter)
      ```

      Gets the instances as object.

      Parameters:
      :   `trainSet` -


    - #### TrainTestInputManager

      ```
      public TrainTestInputManager(int trainArffRecordId,
                           DbManagers dbMan,
                           TrainTestExperimenter trainTestExperimenter)
      ```


    - #### TrainTestInputManager

      ```
      public TrainTestInputManager(int trainArffRecordId,
                           int testArffRecordId,
                           DbManagers dbMan,
                           TrainTestExperimenter experimenter)
      ```
  - ### Method Detail


    - #### checkTestArffRecord

      ```
      public void checkTestArffRecord()
      ```


    - #### getTestArffRecord

      ```
      public MulanArffRecord getTestArffRecord()
      ```


    - #### getTestDataSet

      ```
      public IdMultiLabelInstances getTestDataSet()
      ```


    - #### setTestArffRecord

      ```
      public void setTestArffRecord(int testArffId,
                           DbManagers dbMan)
      ```


    - #### setTestDataSet

      ```
      public void setTestDataSet(IdMultiLabelInstances testSet)
      ```


- Overview
- Package
- Class
- Use
- Tree
- Deprecated
- Index
- Help

- Prev Class
- Next Class

- Frames
- No Frames

- All Classes

- Summary:
- Nested |
- Field |
- Constr |
- Method

- Detail:
- Field |
- Constr |
- Method
